# Microtesla magnetic therapy for cognitive impairment in long COVID: a randomized pilot study

**DOI:** 10.1093/braincomms/fcag364

**Published:** 2026-09-28

**Authors:** Alexandra Canori, Eric Watson, Devanshi Patel, Arianna Fiorentino, Christopher Santiago, David Maltz, Blake Gurfein, David Putrino, Jacqueline Becker

**Affiliations:** Department of Rehabilitation and Human Performance, Icahn School of Medicine at Mount Sinai, New York, NY 10029, USA; Department of Rehabilitation and Human Performance, Icahn School of Medicine at Mount Sinai, New York, NY 10029, USA; Department of Rehabilitation and Human Performance, Icahn School of Medicine at Mount Sinai, New York, NY 10029, USA; Department of Rehabilitation and Human Performance, Icahn School of Medicine at Mount Sinai, New York, NY 10029, USA; Department of Rehabilitation and Human Performance, Icahn School of Medicine at Mount Sinai, New York, NY 10029, USA; Fareon, Inc., San Francisco, CA 94107, USA; Fareon, Inc., San Francisco, CA 94107, USA; Department of Rehabilitation and Human Performance, Icahn School of Medicine at Mount Sinai, New York, NY 10029, USA; Department of Medicine, Icahn School of Medicine at Mount Sinai, New York, NY 10029, USA

**Keywords:** long COVID, cognitive impairment, executive function, magnetic field therapy

## Abstract

Cognitive impairment is common in post-acute sequelae of severe acute respiratory syndrome coronavirus 2 infection (long COVID) and substantially affects function and quality of life. Evidence suggests that sustained neuroinflammation, cerebrovascular dysfunction and mitochondrial impairment contribute to cognitive symptoms. Microtesla Magnetic Therapy (MMT) is a transcranial, low-amplitude radiofrequency magnetic field intervention that has shown anti-inflammatory and neuroprotective effects in preclinical models, suggesting potential value in long COVID and other neurological disorders. This is the first randomized controlled trial of MMT for long COVID cognitive impairment. We evaluated the feasibility (primary) and safety (secondary) of at-home MMT in individuals with objective cognitive impairment from long COVID. Thirty participants were randomized 2:1 to active or sham MMT and self-administered 15-min treatments at home twice weekly for 4 weeks, with remote monitoring, using a head-worn device delivering a non-thermal radiofrequency magnetic field to the whole brain. Feasibility was defined as completing at least 80% of prescribed treatments and all study visits. Safety was assessed by adverse events. Cognitive function and self-reported mood and quality of life were exploratory outcomes assessed at baseline, Week 4 (post-treatment) and Week 8 (follow-up). Feasibility was high: all participants who completed the study adhered to 100% of treatments, and usability ratings were strong. No device-related adverse events occurred. Compared with sham, active MMT participants showed significantly greater improvement from baseline to Week 8 in Wechsler Adult Intelligence Scale Digit Span Sequencing (*P* = 0.026), Hopkins Verbal Learning Test–Revised Total Recall (*P* = 0.044) and Delis-Kaplan Executive Function System Color Naming (*P* = 0.049). Other measures of attention, processing speed and executive function did not differ significantly between groups. Emotional well-being on the 36-Item Short Form Survey improved significantly more with active MMT at Week 8 (*P* = 0.017). Depression and anxiety decreased in both groups; the anxiety reduction was greater with active treatment in the linear mixed-effects model (week × treatment interaction, *P* = 0.038) but not in the between-group change score comparison (*P* = 0.057). At-home MMT was feasible, safe and well tolerated in individuals with cognitive impairment from long COVID. These preliminary, exploratory findings showed nominally significant between-group differences favouring active treatment on selected cognitive measures and emotional well-being and require confirmation in larger, adequately powered trials.

## Teaser

Canori *et al*. report that at-home Microtesla Magnetic Therapy was feasible, safe and well tolerated in 30 adults with cognitive impairment from long COVID. Exploratory outcomes favoured active treatment over sham in working memory, verbal learning, processing speed and emotional well-being, with the largest differences at follow-up.

## Graphical Abstract

**Graphical Abstract:**
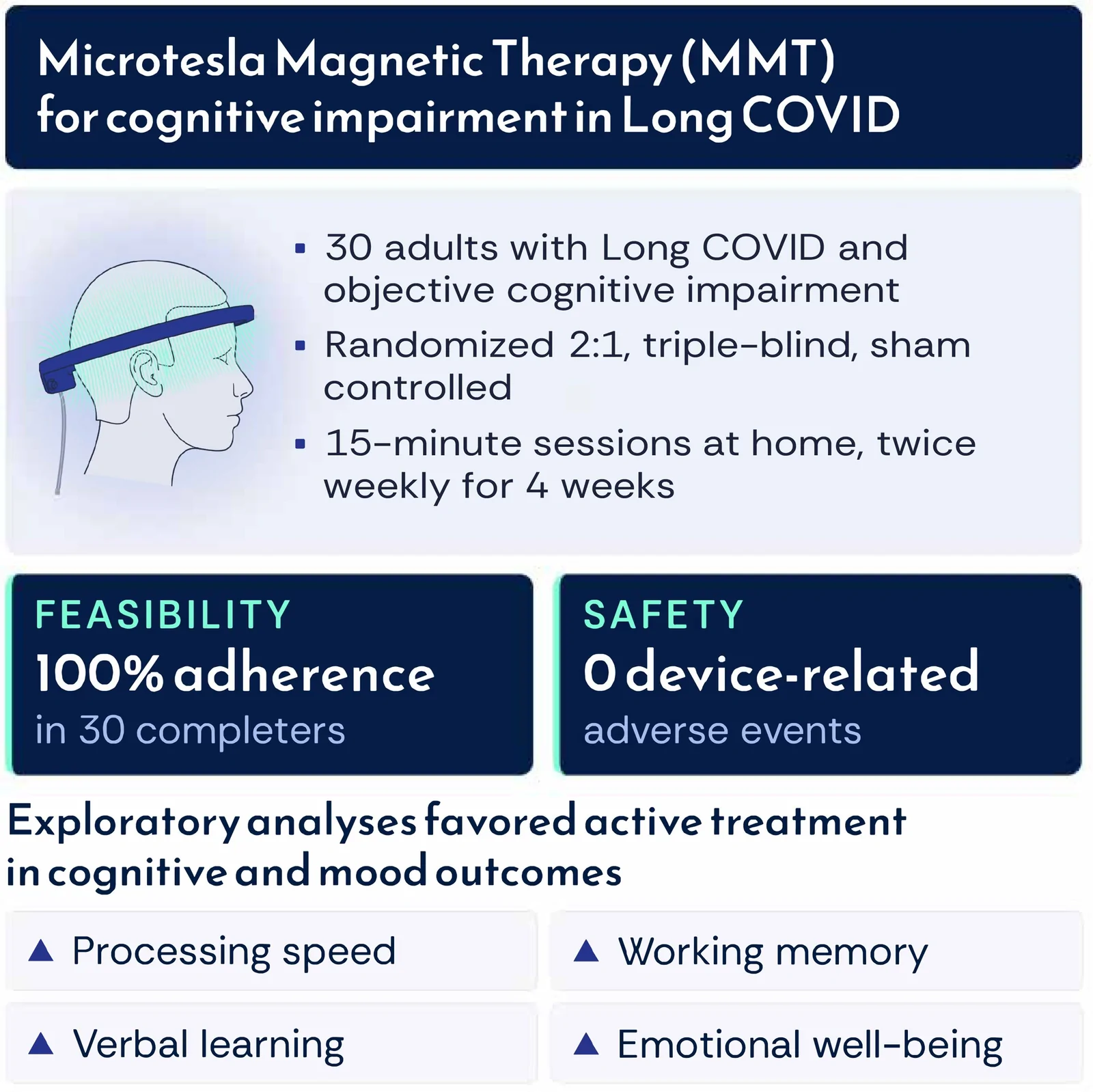

## Introduction

Approximately 10–26% of patients infected with SARS-CoV-2 develop post-acute sequelae of COVID-19, or long COVID (LC),^1^ a condition characterized by symptoms including fatigue, mood changes, sleep disruption and cognitive impairment that persist at least 3 months following acute infection.^2^ The Centers for Disease Control and Prevention estimates LC affects 6.4% of adults in the USA, which is ∼17 million individuals.^3^ Multiple pathophysiologic mechanisms have been implicated in the development of LC, such as persistent peripheral and central nervous system immune activation, incomplete viral clearance or latent viral reservoirs, immune cell exhaustion, vascular and endothelial injury, gut dysbiosis, autoantibody production and dysregulated autonomic signalling.^4-6^ The societal burden of LC is substantial in the USA due to lost productivity, increased healthcare utilization and reduced quality of life,^7^ with estimated annual costs of ∼$200 billion.^8^

Cognitive impairment is among the most prevalent and disabling features of LC, with estimates suggesting that 20–30%, or ∼3–5 million, individuals with LC experience persistent cognitive difficulties.^9,10^ Emerging evidence implicates neuroinflammation as a key underlying driver of cognitive impairment.^11,12^ Specifically, neuroimmune activation and subsequent oxidative stress may perturb mitochondrial bioenergetics and promote a self-perpetuating cycle of energy failure and chronic neuroinflammation, providing a plausible biological basis for persistent cognitive symptoms.^13^ Supporting this framework, several neuroimaging studies have demonstrated microglial activation and elevated inflammatory markers in individuals with LC-related cognitive symptoms.^14,15^ The neurocognitive profile in LC is particularly notable for deficits in executive functioning, attention and processing speed,^16^ which appear to persist over time.^17^ Despite growing mechanistic insight, no therapeutic interventions have demonstrated targeted efficacy.

Low-amplitude magnetic field interventions have demonstrated safety and tolerability, with evidence of rapid anti-inflammatory effects in post-surgical clinical studies^18^ and animal models of neuroinflammation.^19^ Building on this foundation, Microtesla Magnetic Therapy (MMT) was developed as a non-invasive approach to deliver carefully tuned radiofrequency magnetic fields to the brain. Recent preclinical studies showed that MMT suppresses NF-κB signalling, reduces neuronal reactive oxygen species and decreases glial activation and lesion burden *in vivo*, producing sustained anti-inflammatory and neuroprotective effects.^20^ These findings suggest MMT engages redox-sensitive inflammatory networks and modulates neuroimmune activation, pathways increasingly recognized as central drivers of cognitive dysfunction. As a result, MMT represents a promising non-pharmacologic strategy to address a significant unmet clinical need by directly targeting neuroinflammatory biology.

Based on the non-invasive, low-amplitude nature of MMT and compelling mechanistic evidence, the present study evaluated the feasibility of at-home MMT as a therapeutic intervention for individuals with objective cognitive impairment from LC. Our objectives were to (i) assess treatment feasibility and acceptability in this population, (ii) evaluate the safety and tolerability and (iii) collect preliminary efficacy data to inform the design of larger clinical trials. We hypothesized that MMT would be feasible and safe in individuals with objective cognitive impairment from LC, and that active treatment would demonstrate greater improvements in cognitive function and mood symptoms compared with sham treatment.

## Materials and methods

### Study design

A prospective, triple-blind, randomized controlled pilot trial of 30 participants with LC and objective cognitive impairment was conducted to evaluate the feasibility, safety and preliminary efficacy of MMT. Participants were randomized in a 2:1 ratio to receive either an active or sham device. Participants, investigators and outcome assessors were blinded to group allocation. Device assignment was performed by an independent study engineer who pre-programmed active and sham devices prior to delivery to the study site, ensuring allocation concealment. Participants were asked in a post-intervention survey to indicate whether they believed they had received an active or sham device. Blinding integrity was assessed using the index of Petroff *et al*.^21^ The study protocol was approved by the Program for the Protection of Human Subjects at the Icahn School of Medicine at Mount Sinai (STUDY-24-01276) and registered on ClinicalTrials.gov (NCT06739668). All participants provided written informed consent prior to participation.

### Study participants

The following inclusion criteria were used to determine participant eligibility: (i) age 18 years or older, (ii) English speaking, (iii) SARS-CoV-2 infection documented by laboratory nucleic acid amplification test or antibody test ≥6 months from screening, (iv) experiencing symptoms ≥6 months that meet LC diagnostic criteria^2^ (defined as the continuation or new onset of one or more symptoms, including fatigue, post-exertional malaise, cognitive dysfunction and mood or sleep disturbance, present for at least 6 months following confirmed SARS-CoV-2 infection and not explained by an alternative diagnosis),^2^ (v) objective cognitive impairment (defined by ≥1 standard deviation below the normative mean) on at least one measure of executive functioning (WAIS-IV Digit Span, D-KEFS Color–Word Interference Test, D-KEFS Verbal Fluency, or Trail Making Test Part B), (vi) individuals of childbearing age agreeing to use a highly effective form of birth control and (vii) willing and able to provide informed consent and attend all study visits.

Exclusion criteria were determined by self-report or review of electronic health record and included pre-existing cognitive impairment, current use of immunomodulatory medications, active psychiatric or neurological disorders (defined as a current, unstable or inadequately treated DSM-5 diagnosis or neurologic disorder identified by self-report), cranially implanted devices or pacemakers, severe head injury within 12 months, SARS-CoV-2 reinfection or vaccination within 30 days prior to the baseline (Visit 1) assessment, diagnosis of immune/autoimmune condition prior to SARS-CoV-2 infection, change in anti-depressant or other psychoactive medication within 90 days, BrainCheck assessment within the last 6 months, febrile at the time of enrolment visit, enrolment in another clinical trial within 90 days of study period, inability to achieve appropriate positioning of the study device and pregnancy.

### Intervention

Participants randomized to the active group received treatment with the MMT device, which delivers a low-amplitude non-thermal radiofrequency biphasic magnetic field at 27.12 MHz via a head-worn applicator (Fig. 1). Sham devices were visually identical and had the same indicator lights and sounds, but emitted no magnetic field. Participants were trained to self-administer treatments at home twice weekly for 15-min sessions, separated by at least 70 h, a time interval informed by previous *in vivo* studies.^20^ After each treatment session, the MMT device entered a 70-h lockout period to prevent participants from retreating before the next indicated treatment. Due to the high frequency and non-thermal specification of the waveform, active MMT did not generate any perceptible sensation to participants.

**Figure 1:**
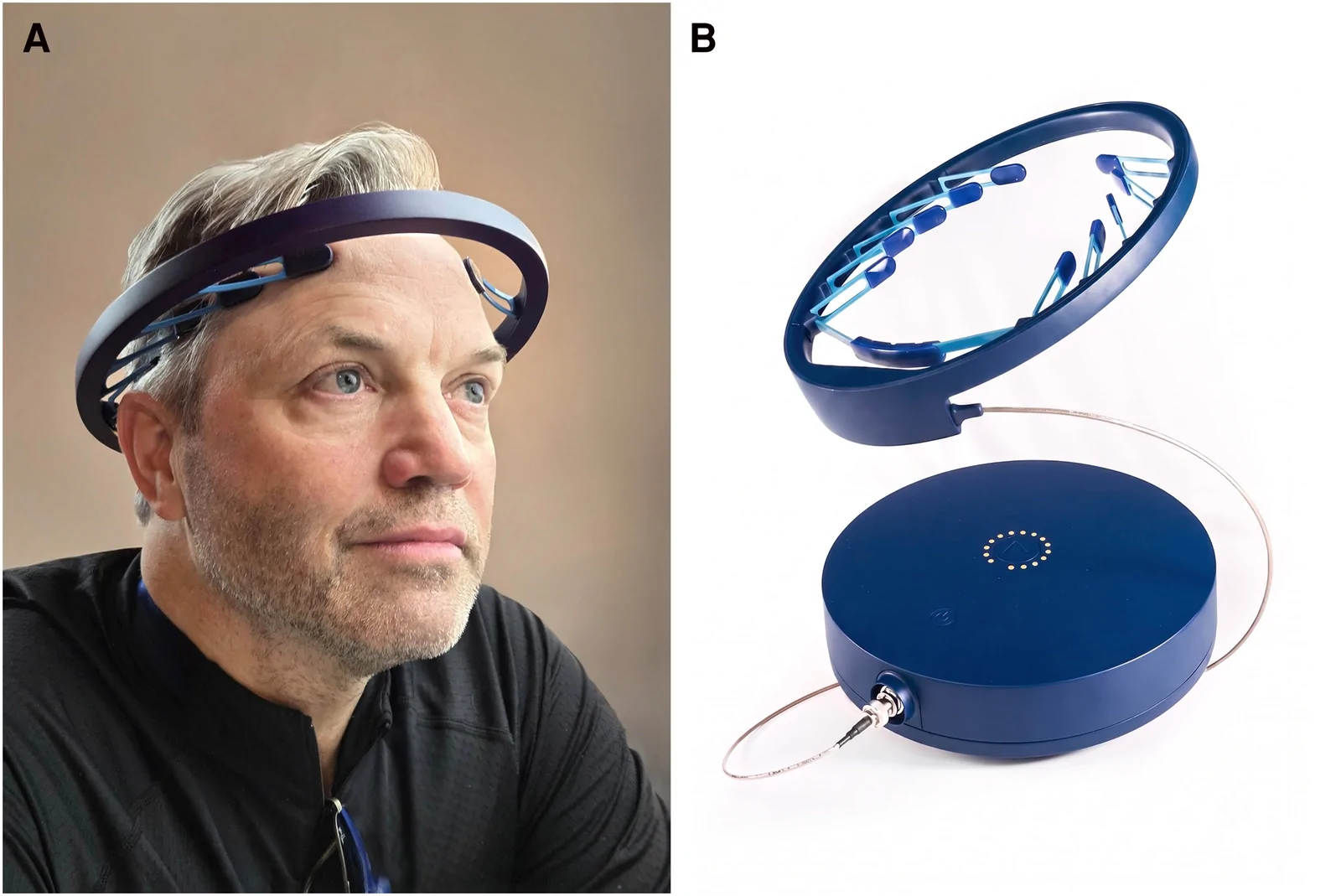
**Device**. (**A**) Fareon Restore Headset positioned on head for therapy; (**B**) Fareon Restore Delivery System.

### Study outcomes

The primary outcome was feasibility, defined as the proportion of participants completing at least 80% of scheduled treatments and all three study visits. Additional feasibility measures included Device Usability Ratings (Supplementary Table S1) and completeness of data collection.

The prespecified secondary outcome was safety. Cognitive performance and self-reported symptom (mood, fatigue and quality of life) changes were exploratory efficacy outcomes. Participants completed a structured weekly safety questionnaire in REDCap that captured the presence, onset, duration and severity of any new or worsening symptoms; investigators additionally elicited adverse events through open-ended questioning and clinical evaluation at each in-person visit. Adverse events were reviewed by the study team, graded for severity and adjudicated for relatedness to the device and treatment according to ISO 14155 criteria.

Objective cognitive performance was assessed with a standardized neurocognitive battery across domains, including attention, processing speed, language, memory and executive functioning. Measures included the Wechsler Adult Intelligence Scale Fourth Edition (WAIS-IV) Digit Span,^22^ Hopkins Verbal Learning Test–Revised (HVLT-R),^23^ Delis-Kaplan Executive Function System (D-KEFS) Color–Word Interference Test,^24^ D-KEFS Verbal Fluency Test,^24^ Trail Making Test (TMT) Parts A & B,^25^ Ruff 2 & 7,^26^ Brief Visuospatial Memory Test (BVMT),^27^ Rey Complex Figure Test (RCFT),^28^ Symbol Digit Modalities Test (SDMT),^29^ and Multilingual Naming Test (MINT).^30^ All assessments were administered in a fixed order by trained staff to minimize order effects and provide consistency across participants.

Mood and quality of life outcomes were assessed using standardized instruments including the Patient Health Questionnaire 9 (PHQ-9),^31^ Generalized Anxiety Disorder 7 (GAD-7),^32^ DePaul Symptom Questionnaire Post-Exertional Malaise (DSQ-PEM),^33^ PROMIS Fatigue Short Form-7a,^34^ and 36-Item Short Form Health Survey (SF-36).^35^ A description of each of these measures is provided in Table 1.

**Table 1:** Outcome measures and description of domains assessed

Measure	Domains and subdomains
**Neurocognitive measures**	
Wechsler Adult Intelligence Scale Fourth Edition (WAIS-IV) Digit Span Forward	Basic auditory attention
WAIS-IV Digit Span Backward (DSB)	Working memory
WAIS-IV Digit Span Sequencing (DSS)	Working memory and executive attention
Hopkins Verbal Learning Test–Revised (HVLT-R)	Verbal learning, delayed recall and retention
Trail Making Test A (TMT A)	Divided attention and graphomotor speed
Trail Making Test B (TMT B)	Cognitive flexibility and executive functioning
Delis-Kaplan Executive Function System (D-KEFS) Verbal Fluency	Language, initiation/fluency and executive control
D-KEFS Color–Word Interference	Processing speed and visuomotor function
Ruff 2 & 7	Sustained and selective attention, inhibition
Brief Visuospatial Memory Test (BVMT)	Visuospatial memory
Rey Complex Figure Test (RCFT)	Visuospatial ability
Symbol Digit Modalities Test (SDMT)	Processing speed and working memory efficiency
Multilingual Naming Test (MINT)	Language and dysnomia
**Mood and quality of life measures**	
Patient Health Questionnaire 9 (PHQ-9)	Presence of depressive symptoms over the past 2 weeks
Generalized Anxiety Disorder 7 (GAD-7)	Presence of anxiety symptoms over the past 2 weeks
DePaul Symptom Questionnaire Post-Exertional Malaise (DSQ-PEM)	Post-exertional malaise, including symptom exacerbation after physical or cognitive exertion, fatigue recovery time and functional impact
PROMIS Fatigue Short Form-7a	Severity and impact of fatigue
36-Item Short Form Health Survey (SF-36)	General health-related quality of life across eight domains: physical functioning, role limitations (physical), bodily pain, general health, vitality, social functioning, role limitations (emotional) and mental health
**Feasibility measures**	
Device Usability Ratings	Assessment of device usability and user experience

*Abbreviations:* WAIS-IV, Wechsler Adult Intelligence Scale, Fourth Edition; HVLT-R, Hopkins Verbal Learning Test–Revised; TMT, Trail Making Test; D-KEFS, Delis-Kaplan Executive Function System; BVMT, Brief Visuospatial Memory Test; RCFT, Rey Complex Figure Test; SDMT, Symbol Digit Modalities Test; MINT, Multilingual Naming Test; PHQ-9, Patient Health Questionnaire-9; GAD-7, Generalized Anxiety Disorder-7; DSQ-PEM, DePaul Symptom Questionnaire-Post-Exertional Malaise; PROMIS, Patient-Reported Outcomes Measurement Information System; SF-36, 36-Item Short Form Health Survey.

### Study procedures

Participants attended three in-person visits (Week 0, Week 4, Week 8). At Visit 1, participants completed informed consent, medical history review and baseline cognitive and self-report assessments. Participants were trained to use the study device and self-administered the first 15-min treatment under the supervision of clinical staff. They were instructed to continue treatments twice per week at home, separated by at least 70 h for the 4-week duration of the treatment period. Each treatment was scheduled as a video visit with the study team, and a team member visually confirmed the accurate positioning of the device and administration of the treatment. Participants returned to the study site for Visits 2 and 3 and repeated all assessments conducted at baseline to evaluate treatment effects and post-treatment persistence (Fig. 2). All visits were scheduled at the same time of day to reduce diurnal variability in performance measures. For cognitive measures with established alternate forms (e.g. HVLT-R and BVMT), parallel versions were administered across visits to reduce practice effects.

**Figure 2:**
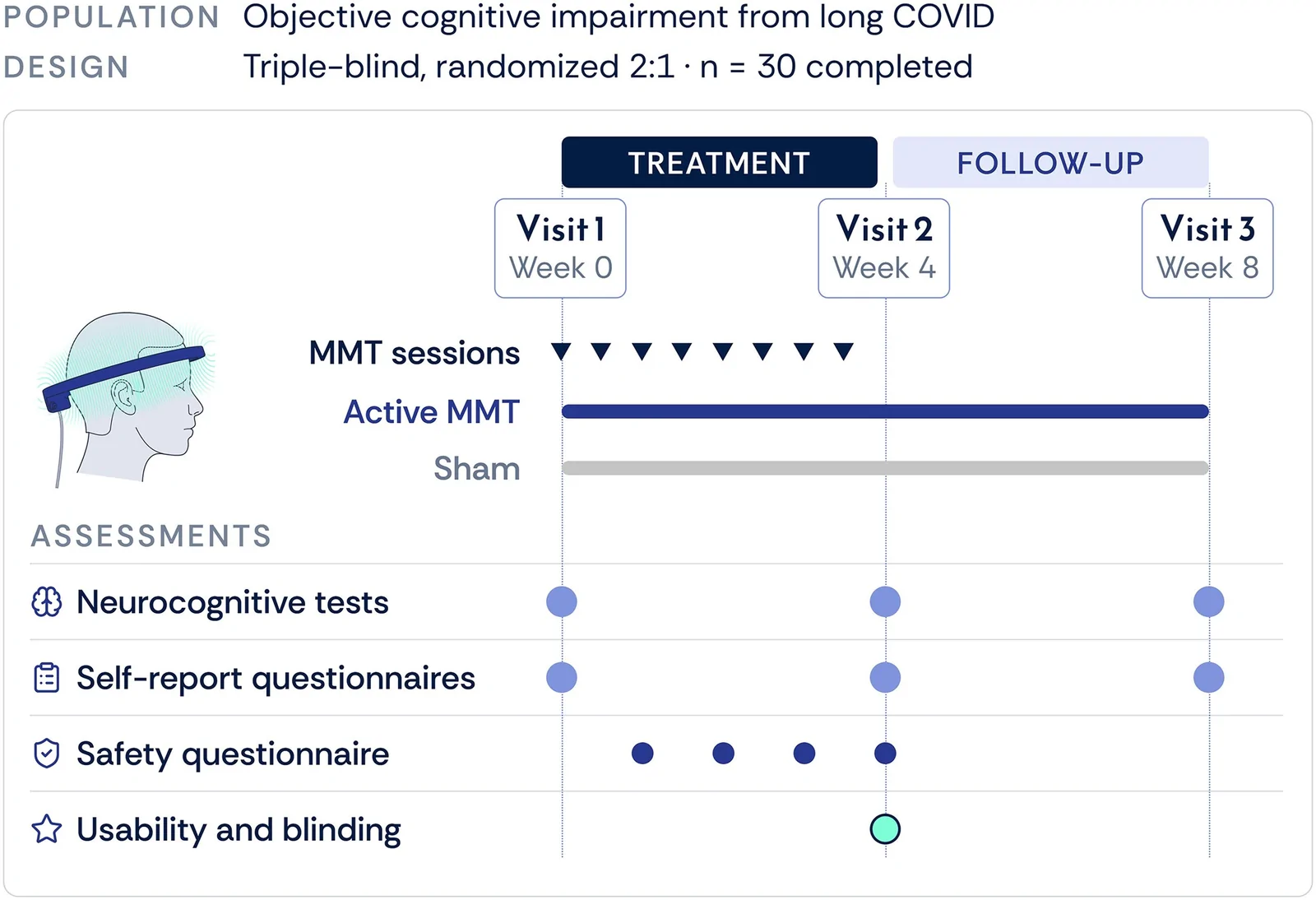
Study design and schedule of procedures.

### Data management

The study was monitored by the principal investigator and study staff. All data were entered into a 21 CFR 11-compliant electronic data capture system. Treatment adherence, device logs and questionnaire completion were monitored weekly, and self-report safety questionnaires were administered weekly to participants during the treatment period and reviewed by the study team. Data quality checks were performed regularly to ensure accuracy and completeness.

### Statistical analysis

#### Feasibility and safety analyses

Feasibility was assessed by the proportion of participants completing at least 80% of prescribed treatments and all scheduled visits. Device usability ratings and responses were summarized. All adverse events and complications occurring during the treatment and follow-up periods were documented and summarized by treatment group.

#### Neurocognitive and self-report measures

Neurocognitive instruments and self-report questionnaires were used to evaluate initial efficacy trends between treatment groups from baseline (Week 0) to post-treatment (Week 4) and from baseline to follow-up (Week 8). We emphasize that for efficacy outcomes, the study was exploratory and not statistically powered to detect prespecified effect sizes for each endpoint. Clinically meaningful change for neurocognitive assessments of cognitive function was set to 0.5 standard deviations.^36^ Clinically meaningful change for PHQ-9 and GAD-7 was interpreted as 2 points, aligning with previous studies.^37^ We fit linear mixed-effects models for all longitudinal outcomes, incorporating treatment, time and treatment × time effects. To simplify reporting of the large number of outcomes, the assessments were also evaluated by comparing the average change from baseline between groups using two-sided *t*-tests assuming unequal variances, with *P*-values reported for directional interpretation. Confidence intervals for the differences between treatment groups are provided for assessment of the observed differences. Cohen’s d was calculated as a measure of effect size for select measures. Analyses were conducted using complete-case data; imputation was not used for missing values. Statistical analysis was performed using JMP Pro software (version 19.0).

## Results

### Participant demographics

A total of 65 participants were recruited and screened; 32 participants failed screening, and 33 were enrolled between December 2024 and October 2025. Reasons for screening exclusion were absence of objective cognitive impairment, current use of immunomodulatory medication and a pre-existing serious immune diagnosis (Fig. 3). The number of participants on psychoactive medication at baseline by group is reported in Supplementary Table S2. To preserve the randomization allocation and ensure that 30 participants completed all study procedures, screening exceeded the target sample size to account for anticipated attrition. Three study participants were withdrawn from the study after enrolment for the following reasons: (i) COVID reinfection during the study period, which per protocol required withdrawal; (ii) two consecutive video visits missed, which per protocol required withdrawal; and (iii) participant injured their ankle and withdrew to pursue surgical intervention. In total, 30 participants with LC-related cognitive impairment completed all trial activities and were analysed (active = 20; sham = 10). Groups were comparable at baseline with respect to age, education, sex, ethnicity and time since SARS-CoV-2 infection (Table 2). Differences in race were observed between groups; however, subgroup sizes were small, limiting interpretability. Supplementary Table S3 summarizes medical history by treatment group, including neurological and psychiatric diagnoses and concomitant medications. The proportion of participants classified as cognitively impaired on each neuropsychological test is reported in Supplementary Table S4, along with the baseline between-group comparison for each measure.

**Figure 3:**
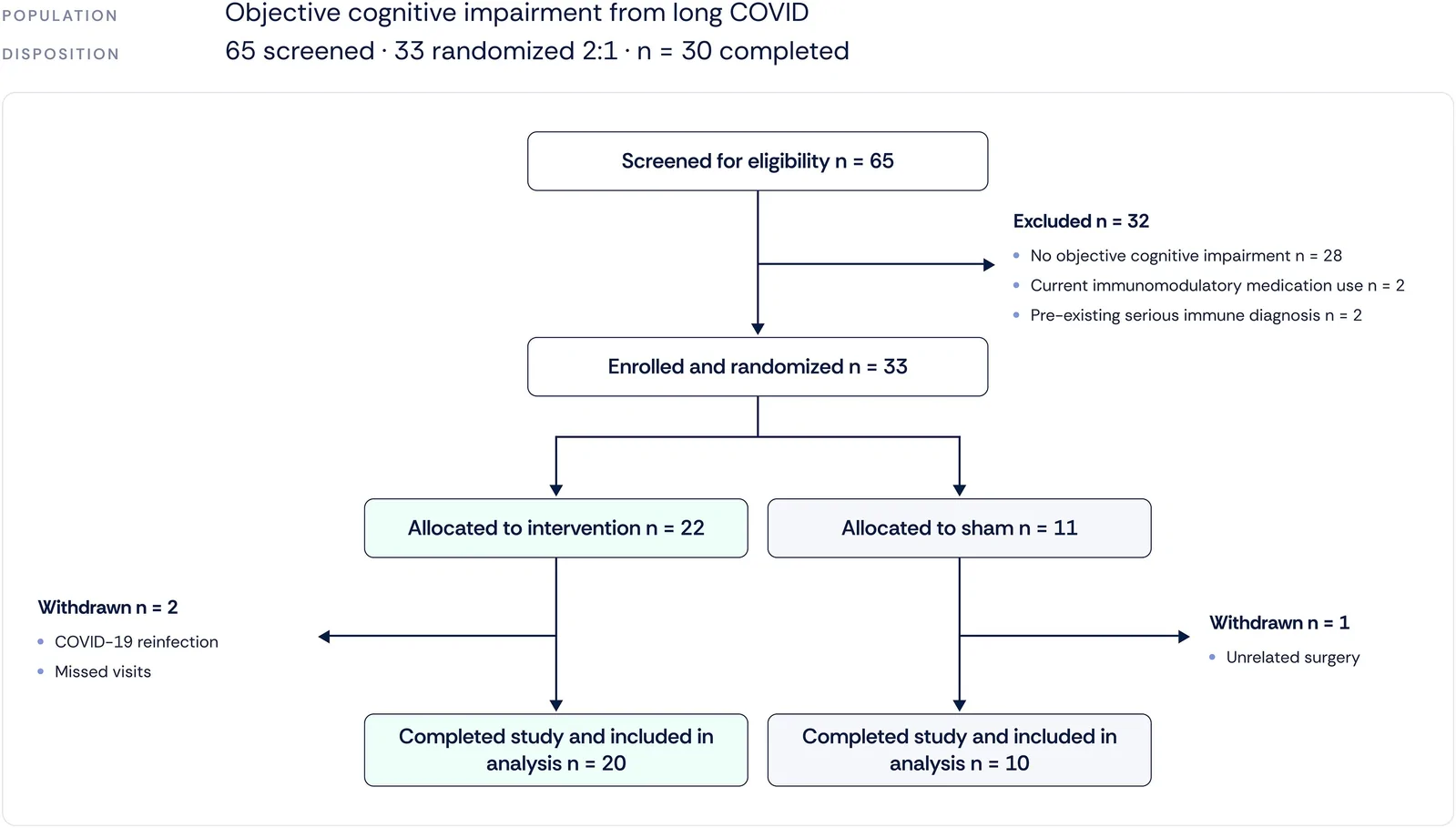
CONSORT flow diagram.

**Table 2:** Baseline demographic and clinical characteristics of study participants

Variable	Treatment			*P*-value
	Sham (*N* = 10)	Active (*N* = 20)	All (*N* = 30)	
Age	48.5 (11.7)	47.7 (13.9)	47.9 (13.0)	0.870 *t*-test
Sex				0.675 Fisher’s exact
Female	8 (80.0%)	13 (65.0%)	21 (70.0%)	
Male	2 (20.0%)	7 (35.0%)	9 (30.0%)	
Education (in years)	17.9 (2.7)	17.5 (2.4)	17.6 (2.5)	0.648 *t*-test
Race				0.011 Fisher’s exact
Asian	1 (10.0%)	2 (10.0%)	3 (10.0%)	
Black	2 (20.0%)	1 (5.0%)	3 (10.0%)	
White	3 (30.0%)	16 (80.0%)	19 (63.3%)	
Other	3 (30.0%)	0 (00.0%)	3 (10.0%)	
Ethnicity				1.0 Fisher’s exact
Hispanic or Latino	2 (20.0%)	3 (15.0%)	5 (16.7%)	
Non-Hispanic or Latino	8 (80.0%)	17 (85.0%)	25 (83.3%)	
Months since SARS-CoV-2 infection	38.9 (12.9)	45.6 (15.7)	43.4 (15.0)	0.513 *t*-test

Data are presented as mean (SD) for continuous variables and *n* (%) for categorical variables. *P*-values are from independent samples *t*-tests (continuous) or Fisher’s exact tests (categorical). SD, standard deviation.

### Feasibility and safety

This study aimed to determine the feasibility of applying the MMT device for treatment at home. All 30 participants who completed the study received all eight treatments in accordance with the protocol, although one participant who did not complete the study was withdrawn due to missing two consecutive visits (i.e. 100% adherence). Using the full randomized sample (*N* = 33), 90.9% completed at least 80% of treatments and all study visits. Correct positioning of the device on the head and completion of the full 15-min treatment duration were confirmed by study staff. Two adverse events occurred in the active treatment group: an ankle injury and SARS-CoV-2 reinfection. Both adverse events were mild and deemed unrelated to treatment. No serious adverse events occurred during this study.

### Linear mixed-effects models

Linear mixed-effects models (Supplementary Table S5) identified a significant week × treatment interaction for GAD-7 (F(2, 56) = 3.48, *P* = 0.038) and the SF-36 Emotional Well-Being subscale (F(2, 56) = 5.86, *P* = 0.005). No other outcome showed a significant interaction; D-KEFS Word Reading showed a significant main effect of treatment (F(1, 28) = 4.67, *P* = 0.039) without an interaction, and 15 of the remaining 22 outcomes showed a significant main effect of week, indicating change over time in both groups without a significant between-group difference in the magnitude of change. The difference-score analyses (Table 3) are reported below, beginning with the two outcomes that showed a significant interaction.

**Table 3:** Change from baseline in outcome measures by treatment group at Weeks 4 and 8

Outcome	Week	Sham, *N*	Active, *N*	Sham, Mean (SD)	Active, Mean (SD)	Active—Sham (95%CI)	*t* Ratio	DF	*P*-value
Digit Span Forward	4 ΔBL	10	20	1.10 (5.63)	4.30 (7.60)	3.20 (−1.9, 8.28)	1.30	23.6	0.2058
	8 ΔBL	10	20	3.20 (6.00)	3.90 (7.11)	0.70 (−4.4, 5.85)	0.28	21.2	0.7801
Digit Span Backward	4 ΔBL	10	20	3.80 (9.67)	3.00 (7.12)	−0.8 (−8.2, 6.59)	−0.23	14.0	0.8198
	8 ΔBL	10	20	2.70 (4.83)	5.65 (8.83)	2.95 (−2.2, 8.07)	1.18	27.6	0.2473
Digit Span Sequencing	4 ΔBL	10	20	−0.5 (4.14)	1.30 (7.35)	1.80 (−2.5, 6.11)	0.86	27.4	0.3992
	8 ΔBL	10	20	−2.1 (4.12)	3.30 (8.49)	5.40 (0.68, 10.1)	2.34	28.0	0.0264*
HVLT-R Total Recall	4 ΔBL	10	20	−4.1 (12.3)	1.34 (13.9)	5.47 (−4.9, 15.8)	1.10	20.4	0.2844
	8 ΔBL	10	20	−4.4 (9.31)	4.62 (13.6)	9.00 (0.28, 17.7)	2.13	25.0	0.0436*
HVLT-R Delayed Recall	4 ΔBL	9	20	1.75 (11.0)	0.18 (15.2)	−1.6 (−12, 8.83)	−0.31	21.2	0.7577
	8 ΔBL	8	20	1.25 (10.5)	1.44 (14.4)	0.20 (−10, 10.5)	0.04	17.9	0.9686
D-KEFS Color Naming	4 ΔBL	10	20	1.80 (5.67)	5.85 (14.4)	4.05 (−3.5, 11.6)	1.10	27.1	0.2820
	8 ΔBL	10	20	3.70 (5.44)	11.1 (14.1)	7.40 (0.05, 14.8)	2.06	27.0	0.0487*
D-KEFS Word Reading	4 ΔBL	10	20	3.70 (12.9)	8.45 (13.3)	4.75 (−5.8, 15.3)	0.94	18.6	0.3590
	8 ΔBL	10	20	7.90 (12.5)	9.10 (14.8)	1.20 (−9.5, 11.9)	0.23	21.1	0.8182
D-KEFS Inhibition	4 ΔBL	10	20	7.50 (9.03)	12.4 (15.1)	4.90 (−4.2, 14.0)	1.11	26.9	0.2774
	8 ΔBL	10	20	14.3 (11.5)	14.7 (16.3)	0.35 (−10, 11.0)	0.07	24.4	0.9464
D-KEFS Inhibition/Switching	4 ΔBL	10	20	9.10 (7.84)	9.90 (13.2)	0.80 (−7.1, 8.71)	0.21	27.0	0.8371
	8 ΔBL	10	20	11.5 (12.3)	12.7 (13.4)	1.15 (−9.1, 11.4)	0.23	19.6	0.8170
D-KEFS Letter Fluency	4 ΔBL	10	20	2.70 (8.06)	5.35 (7.98)	2.65 (−3.9, 9.18)	0.85	17.9	0.4054
	8 ΔBL	10	20	5.00 (6.53)	6.15 (8.71)	1.15 (−4.7, 7.02)	0.41	23.4	0.6891
D-KEFS Category Switching	4 ΔBL	10	20	1.10 (12.3)	0.95 (8.64)	−0.2 (−9.5, 9.19)	−0.03	13.6	0.9729
	8 ΔBL	10	20	3.60 (7.21)	5.10 (8.84)	1.50 (−4.8, 7.76)	0.50	21.8	0.6243
Trail A	4 ΔBL	9	20	3.11 (8.94)	6.45 (10.3)	3.34 (−4.6, 11.3)	0.88	17.8	0.3875
	8 ΔBL	9	20	5.11 (13.2)	12.5 (11.7)	7.39 (−3.6, 18.4)	1.45	13.9	0.1704
Trail B	4 ΔBL	9	20	7.11 (6.45)	6.05 (9.86)	−1.1 (−7.4, 5.31)	−0.34	23.0	0.7336
	8 ΔBL	9	20	7.11 (10.2)	8.00 (9.10)	0.89 (−7.6, 9.40)	0.22	14.0	0.8259
Ruff 2&7 Automatic Speed	4 ΔBL	10	20	2.50 (5.60)	5.70 (6.39)	3.20 (−1.5, 7.94)	1.41	20.4	0.1748
	8 ΔBL	10	20	3.90 (7.37)	9.70 (9.22)	5.80 (−0.6, 12.2)	1.86	22.2	0.0756
Ruff 2&7 Controlled Speed	4 ΔBL	10	20	3.90 (8.33)	5.75 (9.34)	1.85 (−5.2, 8.86)	0.55	20.1	0.5882
	8 ΔBL	10	20	3.40 (9.78)	9.00 (9.94)	5.60 (−2.4, 13.6)	1.47	18.4	0.1583
Ruff 2&7 Total Speed	4 ΔBL	10	20	3.00 (7.07)	5.95 (7.75)	2.95 (−3.0, 8.86)	1.04	19.7	0.3097
	8 ΔBL	10	20	3.50 (8.54)	9.45 (8.94)	5.95 (−1.1, 13.0)	1.77	18.9	0.0927
BVMT total recall	4 ΔBL	10	20	5.10 (10.1)	2.35 (14.1)	−2.8 (−12, 6.48)	−0.61	24.2	0.5446
	8 ΔBL	9	20	−1.7 (20.0)	1.50 (11.4)	3.17 (−13, 19.0)	0.44	10.4	0.6659
SDMT	4 ΔBL	10	20	−1.8 (12.8)	4.50 (9.69)	6.30 (−3.5, 16.1)	1.37	14.4	0.1908
	8 ΔBL	10	20	7.10 (6.33)	5.35 (9.57)	−1.8 (−7.8, 4.28)	−0.60	25.5	0.5556
PHQ-9	4 ΔBL	9	19	−1.1 (4.59)	−2.5 (5.53)	−1.4 (−5.5, 2.80)	−0.69	18.8	0.5017
	8 ΔBL	9	20	−2.3 (5.70)	−2.8 (5.16)	−0.4 (−5.2, 4.35)	−0.19	14.2	0.8540
GAD-7	4 ΔBL	10	20	0.20 (4.39)	−2.6 (3.10)	−2.8 (−6.1, 0.59)	−1.77	13.7	0.0988
	8 ΔBL	10	20	−0.1 (3.81)	−3.0 (3.32)	−2.9 (−5.9, 0.10)	−2.05	16.0	0.0574
DSQ-PEM	4 ΔBL	8	19	−1.5 (3.12)	−1.7 (8.69)	−0.2 (−4.9, 4.46)	−0.10	24.7	0.9180
	8 ΔBL	8	19	1.25 (8.14)	−2.3 (6.57)	−3.6 (−11, 3.58)	−1.10	11.0	0.2956
SF-36 Emotional Well-Being	4 ΔBL	10	20	0.40 (15.1)	7.60 (14.1)	7.20 (−4.9, 19.3)	1.26	17.0	0.2260
	8 ΔBL	10	20	−10 (20.3)	9.20 (11.1)	19.2 (4.15, 34.2)	2.79	11.8	0.0167*
PROMIS Fatigue	4 ΔBL	10	20	−2.7 (6.20)	−2.6 (6.11)	0.12 (−4.9, 5.14)	0.05	17.8	0.9622
	8 ΔBL	10	20	−4.6 (8.18)	−1.7 (5.70)	2.86 (−3.3, 9.06)	0.99	13.5	0.3393

*Abbreviations:* ΔBL, change from baseline; SD, standard deviation; CI, confidence interval; DF, degrees of freedom; HVLT-R, Hopkins Verbal Learning Test–Revised; D-KEFS, Delis-Kaplan Executive Function System; BVMT, Brief Visuospatial Memory Test; SDMT, Symbol Digit Modalities Test; PHQ-9, Patient Health Questionnaire-9; GAD-7, Generalized Anxiety Disorder-7; DSQ-PEM, DePaul Symptom Questionnaire-Post-Exertional Malaise; SF-36, 36-Item Short Form Health Survey; PROMIS, Patient-Reported Outcomes Measurement Information System.

^*^ *P* < 0.05.

### Neurocognitive outcomes

Across multiple neurocognitive domains, between-group differences favoured active MMT, with the largest differences observed at the 8-week follow-up (Table 3). Active treatment was associated with larger mean between-group improvements from baseline to Week 8, with small to medium effect sizes observed for working memory (DSS; *d* = 0.73, *P* = 0.026), verbal learning and memory (HVLT-R Total Recall; *d* = 0.73, *P* = 0.044) and processing speed (D-KEFS Color Naming; *d* = 0.62, *P* = 0.049); none of these differences remained significant after false discovery rate (FDR) correction, and none showed a significant week × treatment interaction in the mixed-effects models (Supplementary Table S5). While the active group showed greater improvements than sham on remaining measures of attention, processing speed, inhibition and executive function, these differences were not statistically significant (all *P* > 0.05); the corresponding between-group differences, 95% confidence intervals and effect sizes are reported in Table 3, and individual participant points and group means are displayed in Supplementary Fig. S1. The D-KEFS Category Fluency, RCFT, SDMT and MINT did not show improvements from baseline to 8 weeks.

### Mood and quality of life outcomes

Anxiety (GAD-7) symptoms decreased in both groups; the greater reduction in the active group was significant in the mixed-effects model (Supplementary Table S5) but did not reach statistical significance in the difference-score analysis (*d* = −0.83, *P* = 0.057).

On the SF-36 Emotional Well-Being (Mental Health) subscale, participants receiving active MMT showed a significantly greater improvement from baseline to Week 8 compared with sham, with a large effect size (*d* = 1.31, *P* = 0.017, uncorrected). The emotional well-being difference did not remain significant after FDR correction.

Among the outcomes without a significant week × treatment interaction, depression (PHQ-9) symptoms decreased in both groups over time without a significant between-group difference in the magnitude of change (Table 3). Trending improvements in DSQ-PEM scores were observed in the active treatment group (*d* = −0.50, *P* = 0.296).

### Usability and user experience

Participants reported high usability and satisfaction with the device. Across 12 steps of using the device (from removing the device from the case to putting the device away), two participants indicated a challenge with any step, and three participants indicated a challenge with lifting the device for transport. At least 25/30 participants rated the device as a 4/5 or 5/5 (on a 5-point scale) for ease of use, comfort, reliability and convenience. In addition, 24/30 rated their agreement as a 4/5 or 5/5, indicating they would recommend it to others (Supplementary Table S1).

### Blinding integrity

Blinding integrity was assessed using the Petroff index.^21^ This index is simply the difference in the proportion of those guessing they received the active treatment across the two treatment arms and is appropriate for studies in which the randomization is not 1:1. The index ranges from −1 to 1, with 0 indicating perfect blinding. The method assumes all participants provide a guess; however, one participant in our sample did not provide a guess. Excluding that participant yielded an index = −0.02, and including that participant with an incorrect guess (worst case) yielded an index = −0.05 (Supplementary Table S6). Both estimates support the blinding integrity of this study.

## Discussion

This triple-blind, first-in-human, randomized, sham-controlled study demonstrates that at-home MMT is feasible, safe and well tolerated in individuals with objective cognitive impairment related to LC. Participants showed 100% treatment compliance and high usability ratings, with no device-related adverse events, indicating strong feasibility and an excellent safety profile for at-home implementation.

MMT treatment was also associated with clinically meaningful improvements in cognition and mood at treatment completion and at 8-week follow-up. Nominally significant (uncorrected) between-group differences favouring active treatment were observed on measures of working memory (Digit Span Sequencing), processing speed (D-KEFS Color Naming) and verbal learning and memory (HVLT-R Total Recall); no differences remained statistically significant after correction for multiple comparisons. A nominally significant between-group improvement in emotional well-being (SF-36) was also observed, whereas depression and anxiety symptoms declined in both groups (anxiety reductions were greater in the active group in the mixed-effects model, although the between-group difference in change scores did not reach statistical significance). While this exploratory study was not powered for efficacy conclusions, the pattern of differences across several neurocognitive domains and their persistence at follow-up provide preliminary support for the potential clinical value of MMT in addressing an urgent, unmet need in this population (which will require confirmation in adequately powered trials).

The magnitude of cognitive improvement observed with MMT compares favourably to other interventions studied in LC populations.^38-40^ Cognitive rehabilitation approaches have demonstrated modest benefits in pilot studies, with small-to-medium effect sizes and improvements largely limited to self-reported cognitive functioning.^17,41,42^ Pharmacologic interventions targeting cognition in LC remain largely untested in rigorous trials, and those repurposed from other conditions (e.g. stimulants, cholinesterase inhibitors) lack sufficient evidence specific to post-viral populations.^43,44^ Notably, MMT produced improvements across multiple cognitive measures, including processing speed, attention and memory, suggesting potential for broader cognitive benefit within the very domains impacted in LC. Furthermore, unlike cognitive rehabilitation protocols that require sustained mental effort and may be limited by post-exertional symptom exacerbation, MMT is passive and low-burden, potentially improving accessibility to patients who cannot tolerate effortful interventions. Non-invasive brain stimulation approaches have also been explored in post-COVID populations, including through NIH RECOVER-Neuro^40^ and small trials and case series of transcranial direct current stimulation (tDCS). These studies typically target the dorsolateral prefrontal cortex and have reported preliminary improvements in fatigue and, less consistently, in cognition.^45-47^ MMT differs from tDCS and other neuromodulation techniques in that it is intended to modulate neuroinflammatory and metabolic signalling rather than to directly stimulate or inhibit neuronal firing.

### Mechanistic inferences

The pattern of cognitive improvement observed in this study is consistent with emerging mechanistic models of LC-related cognitive impairment that emphasize persistent neuroinflammation, oxidative stress and mitochondrial impairment. Between-group differences in working memory (DSS), verbal learning and memory (HVLT-R Total Recall), processing speed (D-KEFS Color Naming) and emotional well-being (SF-36) were larger at 8-week follow-up than immediately post-treatment, suggesting persistence of benefit and durability of treatment effects. It is possible that the delay of these effects suggests a mechanism involving gradual resolution of neuroinflammation and restoration of neuronal and glial function, rather than short-term expectancy effects alone.

This is the first time that an MMT device has been used in a clinical population, and therefore mechanistic understanding is still emerging. Recent preclinical work found that brief exposure to MMT suppressed inflammatory signalling in human immune cells, and repeated transcranial MMT attenuated microgliosis, astrogliosis, immune cell infiltration and tissue degeneration in a rat model of Parkinson’s disease neuroinflammation.^20^ In this work, MMT also reduced neuronal oxidative stress and improved intrinsic antioxidant capacity, with neuroprotective effects persisting for up to 48 h following a single exposure.^20^ The clinical changes observed here are consistent with, but do not demonstrate, the ability of MMT to suppress neuroinflammation, improve mitochondrial function and promote improved regulation of neurons and glia, as these are all mechanisms of LC-related cognitive loss that have been previously described.^12,48-50^ However, it should be noted that these mechanistic interpretations remain hypothetical.

MMT is distinct from conventional neuromodulation approaches in several important ways. The intervention delivers low-amplitude, non-thermal radiofrequency magnetic fields to the whole brain and was developed to target neuroinflammatory signalling and cellular metabolic function, rather than directly stimulate or inhibit neuronal firing through focal electrical, magnetic, or ultrasound stimulation. The therapy is designed for self-administration in the home environment using a head-worn device. Automated treatment lockout intervals and remote monitoring supported consistent dosing while minimizing user burden and protocol deviation. These characteristics may enable scalable deployment if efficacy is confirmed, particularly for individuals with chronic illness, fatigue-related disability, or limited access to in-person therapy.

### Limitations and future implications

This study has several limitations. The small sample size limited statistical power, and multiple outcomes were assessed without correction for multiple comparisons. Findings should be interpreted as exploratory. Additional limitations include possible selection bias arising from screening exclusions and post-randomization withdrawals; a highly educated sample (mean education ∼17.6 years), which may limit generalizability; a baseline imbalance in racial composition between groups; and the industry funding and device-company involvement in the study. A longer treatment course and follow-up period may be required to fully evaluate efficacy if MMT acts by modulating neuroinflammation, and future adequately powered trials should incorporate mechanistic endpoints, correction for multiplicity and reporting of the proportion of participants achieving reliable or clinically meaningful change.

Repeated neuropsychological testing can introduce practice effects; however, the sham-controlled design helps minimize this influence, and the domain-specific pattern of improvement along with the greater magnitude of change at delayed follow-up compared with immediately post-treatment suggests that the findings are unlikely to be driven by practice effects alone. Despite these limitations, consistent improvements across neurocognitive domains and mood, the presence of statistically significant and clinically meaningful improvements on certain measures and the excellent feasibility and safety profile provide a strong rationale for future investigation. These findings may inform the design of larger clinical trials to confirm efficacy and clarify mechanisms.

## Conclusions

MMT is a feasible, safe and well-tolerated non-pharmacologic intervention and represents a promising candidate for the treatment of cognitive impairment associated with LC. This study supports the feasibility and safety of at-home MMT and provides preliminary, exploratory signals of benefit in selected cognitive domains and emotional well-being in people with LC. Future larger, adequately powered, mechanistically informed trials will be needed to confirm these findings.

## Supplementary Material

fcag364_Supplementary_Data

## Data Availability

De-identified data and supporting documents will be available upon reasonable request.
