# Supplementary material for: Microtesla magnetic therapy for cognitive impairment in long COVID: a randomized pilot study
Source: Brain Commun. 2026 Sep 28;8(5):fcag364. doi: 10.1093/braincomms/fcag364 (PMC13644367; doi:10.1093/braincomms/fcag364)
Supplement: Supplementary file 1 [file fcag364_supplementary_data.docx]

Supplementary Table S1. Participant-reported device usability and user experience (N = 30)

| **Question** | **1 –**  **Disagree** | **2** | **3** | **4** | **5 –**  **Agree** |
| --- | --- | --- | --- | --- | --- |
| I always knew how much time remained in a therapy session. | 3 | 0 | 0 | 4 | 23 |
| I can rely on the study device to deliver therapy consistently. | 0 | 1 | 4 | 4 | 21 |
| I found the audio indicating therapy helpful. | 0 | 0 | 2 | 7 | 21 |
| I would recommend the study device to others. | 0 | 0 | 6 | 4 | 20 |
| The controls of study device were easy to understand. | 0 | 0 | 2 | 2 | 26 |
| The device instructions were easy to understand. | 0 | 0 | 1 | 5 | 24 |
| The study device was comfortable to wear during therapy. | 0 | 0 | 3 | 4 | 23 |
| The study device was easy to use. | 0 | 0 | 0 | 5 | 25 |
| Therapy with the study device was convenient. | 0 | 0 | 0 | 5 | 25 |

Values are the number of respondents (N = 30) selecting each response on a 5-point Likert scale (1 = Disagree, 5 = Agree).

**Supplementary Table S2. Baseline psychoactive medication use by treatment group**

|  | **Active (n = 20)** | **Sham (n = 10)** | **Total (n = 30)** | **p** |
| --- | --- | --- | --- | --- |
| On any psychoactive medication | 8 (40.0%) | 3 (30.0%) | 11 (36.7%) | 0.702 |
| Not on any psychoactive medication | 12 (60.0%) | 7 (70.0%) | 19 (63.3%) |  |

Values are n (%) of randomized subjects. P-value is from Fisher’s exact test, two-sided.

*Definition of psychoactive medication:* A subject was classified as positive if the baseline medication list included one or more prescription agents whose primary pharmacologic action is on the central nervous system. Qualifying classes were antidepressants, psychostimulants, benzodiazepines, non-benzodiazepine hypnotics, antipsychotics, non-benzodiazepine anxiolytics, centrally acting anticonvulsants, central alpha-2 adrenergic agonists, and barbiturate-containing combination analgesics.

**Supplementary Table S3. Medical history and concomitant medications by treatment group**

**A. Neurological diagnoses**

| **Group** | **Neurological Diagnoses** | **n** |
| --- | --- | --- |
| **Active N = 20** | No history recorded | 14 |
|  | ADHD | 1 |
|  | Mild cognitive decline | 1 |
|  | Possible MCI, migraines | 1 |
|  | Post covid cognitive impairment | 1 |
|  | Seizure | 1 |
|  | Stroke | 1 |
| **Sham N = 10** | No history recorded | 6 |
|  | Neuropathy in head and neck, sudden hearing loss  New Daily Persistent Headache (NDPH) | 1 |
|  | ADHD | 1 |
|  | Migraines | 1 |
|  | MRI showed mild to moderate of atrophy white matter, migraine aura | 1 |

**B. Psychiatric diagnoses**

| **Group** | **Psychiatric Diagnoses** | **n** |
| --- | --- | --- |
| **Active N = 20** | No history recorded | 14 |
|  | Depression | 2 |
|  | Depression, anxiety | 2 |
|  | Mood disorder, depression, anxiety | 1 |
|  | Covid-Depression and Anxiety well controlled with medicines | 1 |
| **Sham N = 10** | No history recorded | 5 |
|  | Depression, Anxiety | 2 |
|  | MDD | 1 |
|  | PTSD | 1 |
|  | Recovered from depression | 1 |

**C. Concomitant medications**

| **Group** | **Medications List per Subject** | **n** |
| --- | --- | --- |
| **Active N = 20** | No medications | 5 |
|  | Adderall, Topamax, Seroquel, Doxepin, Claritin, Pepcid, Diltiazem | 1 |
|  | Albuterol, Buspirone, Dexedrine, Motegrity, Fexofenadine, Rifaximin | 1 |
|  | Amlodipine, Levothyroxine, Lisinopril–hydrochlorothiazide, Oxybutynin, Diclofenac, Flocenin, Tylenol | 1 |
|  | Bacopa monnieri | 1 |
|  | Bupropion XL, Duloxetine, Metformin, Colchicine, Metoprolol Succinate XL, Tizanidine, Vitamin B, ASA, Rosuvastatin, Albuterol, Azelastine, Lisinopril–hydrochlorothiazide, Fluticasone propionate | 1 |
|  | Corlanor, Naltrexone, Omeprazole, Famotidine, Sertraline, Clonazepam, Sumatriptan, Albuterol, Ondansetron, | 1 |
|  | Lexapro, Abilify, Lamictal, Klonopin, lorazapam, midodrine, Mestinon, Armour Thyroid, levothyroxine, naltrexone, fomatadine, Allegra, Adderall | 1 |
|  | Lexapro, Vitassium salt capsules, Vitamin D3 | 1 |
|  | Naltrexone | 1 |
|  | Nortriptyline, Ambien ER, Clonidine, Melatonin, Mestinon, Wegovy, Sumatriptan, Fioricet | 1 |
|  | Valsartan, LDN | 1 |
|  | Valsartan, Naltrexone, Sertraline | 1 |
|  | VitaminD250000, Pepcid | 1 |
|  | Zyrtec | 1 |
| **Sham N = 10** | No medications | 4 |
|  | Ambien, Allegra | 1 |
|  | Gabapentin, Nortriptyline, Cymbalta, Topiramate | 1 |
|  | Naltrexone, Metoprolol, Famotidine, Allegra, Creon, Motegrity, Neurontin | 1 |
|  | Propranolol, Aspirin, Cetirizine, Semaglutide | 1 |

Values are the number of participants (n) reporting each diagnosis or medication regimen within the Active (N = 20) and Sham (N = 10) groups. Medication and diagnosis entries are reproduced as recorded. ADHD, attention-deficit/hyperactivity disorder; MCI, mild cognitive impairment; MDD, major depressive disorder; PTSD, post-traumatic stress disorder; NDPH, new daily persistent headache.

**Supplementary Table S4. Frequency of cognitive impairment at baseline by treatment group**

| **Outcome** | **Sham (N = 10)** | | **Active (N = 20)** | | **Fisher’s exact 2-sided P** |
| --- | --- | --- | --- | --- | --- |
|  | **n impaired** | **%** | **n impaired** | **%** |  |
| Digit Span Forward | 6 | 60% | 11 | 55% | 1.0000 |
| Digit Span Backward | 5 | 50% | 12 | 60% | 0.7055 |
| Digit Span Sequencing | 2 | 20% | 6 | 30% | 0.6821 |
| HVLT-R Total Recall | 4 | 40% | 10 | 50% | 0.7089 |
| HVLT-R Delayed Recall | 2 | 22% | 7 | 35% | 0.6749 |
| D-KEFS Color Naming | 3 | 30% | 7 | 35% | 1.0000 |
| D-KEFS Word Reading | 3 | 30% | 5 | 25% | 1.0000 |
| D-KEFS Inhibition | 7 | 70% | 10 | 50% | 0.4404 |
| D-KEFS Inhibition/Switching | 4 | 40% | 10 | 50% | 0.7089 |
| D-KEFS Letter Fluency | 0 | 0% | 2 | 10% | 0.5402 |
| D-KEFS Category Switching | 1 | 10% | 1 | 5% | 1.0000 |
| D-KEFS Category Fluency | 2 | 20% | 1 | 5% | 0.2512 |
| Trail A | 5 | 56% | 9 | 45% | 0.6999 |
| Trail B | 5 | 56% | 12 | 60% | 1.0000 |
| Ruff 2&7 Automatic Speed | 5 | 50% | 8 | 40% | 0.7055 |
| Ruff 2&7 Controlled Speed | 4 | 40% | 6 | 30% | 0.6904 |
| Ruff 2&7 Total Speed | 3 | 30% | 5 | 25% | 1.0000 |
| BVMT total recall | 5 | 50% | 7 | 35% | 0.4611 |
| SDMT | 6 | 60% | 13 | 65% | 1.0000 |

Cognitive impairment defined as a T-score < 40 (i.e., more than 1 SD below the normative mean) on each neuropsychological test at baseline. n impaired, number of participants meeting the impairment threshold; SD, standard deviation. Values are the number of participants.

**Supplementary Table S5. Linear mixed-effects models of longitudinal cognitive, mood, and fatigue outcomes**

| **Outcome** | **Fixed effect** | **Num df** | **Den df** | **F ratio** | **P-value** |
| --- | --- | --- | --- | --- | --- |
| BVMT total recall | Treatment | 1 | 28.3 | 0.416 | 0.5243 |
|  | Week | 2 | 55.6 | 1.246 | 0.2956 |
|  | Week × Treatment | 2 | 55.6 | 0.416 | 0.6616 |
| D-KEFS Category Fluency | Treatment | 1 | 28.0 | 0.030 | 0.8638 |
|  | Week | 2 | 56.0 | 0.955 | 0.3911 |
|  | Week × Treatment | 2 | 56.0 | 0.961 | 0.3888 |
| D-KEFS Category Switching | Treatment | 1 | 28.0 | 0.046 | 0.8315 |
|  | Week | 2 | 56.0 | 3.025 | 0.0566 |
|  | Week × Treatment | 2 | 56.0 | 0.122 | 0.8856 |
| D-KEFS Color Naming | Treatment | 1 | 28.0 | 0.349 | 0.5593 |
|  | Week | 2 | 56.0 | 6.361 | 0.0032 * |
|  | Week × Treatment | 2 | 56.0 | 1.595 | 0.2121 |
| D-KEFS Inhibition | Treatment | 1 | 28.0 | 0.694 | 0.4118 |
|  | Week | 2 | 56.0 | 19.402 | <0.0001 * |
|  | Week × Treatment | 2 | 56.0 | 0.661 | 0.5203 |
| D-KEFS Letter Fluency | Treatment | 1 | 28.0 | 0.872 | 0.3584 |
|  | Week | 2 | 56.0 | 7.431 | 0.0014 * |
|  | Week × Treatment | 2 | 56.0 | 0.396 | 0.6748 |
| D-KEFS Inhibition/Switching | Treatment | 1 | 28.0 | 1.716 | 0.2009 |
|  | Week | 2 | 56.0 | 15.646 | <0.0001 * |
|  | Week × Treatment | 2 | 56.0 | 0.034 | 0.9670 |
| D-KEFS Word Reading | Treatment | 1 | 28.0 | 4.669 | 0.0394 * |
|  | Week | 2 | 56.0 | 7.562 | 0.0012 * |
|  | Week × Treatment | 2 | 56.0 | 0.602 | 0.5514 |
| Digit Span Backward | Treatment | 1 | 28.0 | 0.108 | 0.7447 |
|  | Week | 2 | 56.0 | 4.004 | 0.0237 * |
|  | Week × Treatment | 2 | 56.0 | 0.792 | 0.4580 |
| Digit Span Forward | Treatment | 1 | 28.0 | 0.686 | 0.4146 |
|  | Week | 2 | 56.0 | 4.681 | 0.0132 * |
|  | Week × Treatment | 2 | 56.0 | 0.964 | 0.3876 |
| DSQ-PEM | Treatment | 1 | 28.2 | 0.185 | 0.6701 |
|  | Week | 2 | 53.3 | 0.460 | 0.6335 |
|  | Week × Treatment | 2 | 53.3 | 0.895 | 0.4145 |
| Digit Span Sequencing | Treatment | 1 | 28.0 | 0.360 | 0.5533 |
|  | Week | 2 | 56.0 | 0.131 | 0.8771 |
|  | Week × Treatment | 2 | 56.0 | 2.661 | 0.0787 |
| GAD-7 | Treatment | 1 | 28.0 | 0.327 | 0.5717 |
|  | Week | 2 | 56.0 | 3.413 | 0.0399 * |
|  | Week × Treatment | 2 | 56.0 | 3.478 | 0.0377 * |
| HVLT-R Delayed Recall | Treatment | 1 | 28.6 | 0.993 | 0.3274 |
|  | Week | 2 | 55.0 | 0.160 | 0.8523 |
|  | Week × Treatment | 2 | 55.0 | 0.193 | 0.8250 |
| HVLT-R Total Recall | Treatment | 1 | 28.0 | 0.157 | 0.6954 |
|  | Week | 2 | 56.0 | 0.248 | 0.7816 |
|  | Week × Treatment | 2 | 56.0 | 1.801 | 0.1745 |
| PHQ-9 | Treatment | 1 | 27.8 | 0.407 | 0.5289 |
|  | Week | 2 | 54.1 | 4.299 | 0.0185 * |
|  | Week × Treatment | 2 | 54.1 | 0.429 | 0.6533 |
| PROMIS Fatigue | Treatment | 1 | 28.0 | 2.321 | 0.1388 |
|  | Week | 2 | 56.0 | 4.092 | 0.0219 * |
|  | Week × Treatment | 2 | 56.0 | 0.930 | 0.4007 |
| Ruff 2&7 Automatic Speed | Treatment | 1 | 28.0 | 1.201 | 0.2824 |
|  | Week | 2 | 56.0 | 12.791 | <0.0001 * |
|  | Week × Treatment | 2 | 56.0 | 2.302 | 0.1094 |
| Ruff 2&7 Controlled Speed | Treatment | 1 | 28.0 | 1.580 | 0.2191 |
|  | Week | 2 | 56.0 | 7.228 | 0.0016 * |
|  | Week × Treatment | 2 | 56.0 | 1.388 | 0.2581 |
| Ruff 2&7 Total Speed | Treatment | 1 | 28.0 | 1.547 | 0.2238 |
|  | Week | 2 | 56.0 | 10.354 | 0.0001 * |
|  | Week × Treatment | 2 | 56.0 | 2.084 | 0.1339 |
| SDMT | Treatment | 1 | 28.0 | 0.376 | 0.5444 |
|  | Week | 2 | 56.0 | 6.456 | 0.0030 * |
|  | Week × Treatment | 2 | 56.0 | 2.698 | 0.0761 |
| SF-36 Emotional Well-Being | Treatment | 1 | 28.0 | 1.237 | 0.2755 |
|  | Week | 2 | 56.0 | 1.474 | 0.2378 |
|  | Week × Treatment | 2 | 56.0 | 5.856 | 0.0049 * |
| Trail A | Treatment | 1 | 28.2 | 1.656 | 0.2086 |
|  | Week | 2 | 55.3 | 9.576 | 0.0003 * |
|  | Week × Treatment | 2 | 55.3 | 1.185 | 0.3135 |
| Trail B | Treatment | 1 | 27.3 | 0.170 | 0.6835 |
|  | Week | 2 | 54.4 | 9.219 | 0.0004 * |
|  | Week × Treatment | 2 | 54.4 | 0.016 | 0.9841 |

Separate linear mixed-effects models were fitted for each outcome with fixed effects of treatment group (active, sham), week (0, 4, and 8), and their interaction (Week × Treatment), and a random effect for participant. Each row reports the Type III test of the corresponding fixed effect. Cognitive outcomes are analyzed as age-adjusted T-scores. Num df and Den df, numerator and denominator degrees of freedom; F ratio, F statistic. * P < 0.05.

**Supplementary Table S6. Assessment of blinding: participant guess of treatment assignment**

| **Actual treatment** | **Do you think that you received active or sham (placebo) therapy?** | | | |
| --- | --- | --- | --- | --- |
|  | **Not answered (not sure)** | **Active** | **Sham** | **All** |
| Active | 1 | 13 | 6 | 20 |
| Sham | 0 | 7 | 3 | 10 |
| All | 1 | 20 | 9 | 30 |

**Supplementary Figure S1. Neurocognitive and self-report outcomes.** Individual participant scores are shown at baseline (week 0), week 4, and week 8 for the sham group (n = 10, gray) and the active treatment group (n = 20, black). Within each time point, error bars indicate the 95% confidence interval for the group mean, and group means are connected across time points by a line. The measure depicted is labeled above each panel. (A) Digit Span Forward; (B) Digit Span Backward; (C) Digit Span Sequencing; (D) HVLT-R Total Recall; (E) HVLT-R Delayed Recall; (F) D-KEFS Color Naming; (G) D-KEFS Word Reading; (H) D-KEFS Inhibition; (I) D-KEFS Inhibition/Switching; (J) D-KEFS Letter Fluency; (K) D-KEFS Category Switching; (L) Trail Making Test Part A; (M) Trail Making Test Part B; (N) Ruff 2&7 Automatic Detection Speed; (O) Ruff 2&7 Controlled Search Speed; (P) Ruff 2&7 Total Speed; (Q) BVMT-R Total Recall; (R) PHQ-9; (S) GAD-7; (T) DSQ-PEM; (U) SF-36 Emotional Well-Being; (V) PROMIS Fatigue; (W) SDMT. Panels A to Q and W depict neurocognitive test scores; panels R to V depict self-report questionnaire scores. HVLT-R, Hopkins Verbal Learning Test-Revised; D-KEFS, Delis-Kaplan Executive Function System; BVMT-R, Brief Visuospatial Memory Test-Revised; PHQ-9, Patient Health Questionnaire 9; GAD-7, Generalized Anxiety Disorder 7; DSQ-PEM, DePaul Symptom Questionnaire Post-Exertional Malaise; SF-36, 36-Item Short Form Health Survey; PROMIS, Patient-Reported Outcomes Measurement Information System; SDMT, Symbol Digit Modalities Test.
